# A Complete Pipeline for Untargeted Urinary Volatolomic Profiling with Sorptive Extraction and Dual Polar and Nonpolar Column Methodologies Coupled with Gas Chromatography Time-of-Flight Mass Spectrometry

**DOI:** 10.1021/acs.analchem.2c02873

**Published:** 2023-01-05

**Authors:** Qing Wen, Antonis Myridakis, Piers R. Boshier, Simone Zuffa, Ilaria Belluomo, Aaron G. Parker, Sung-Tong Chin, Stephanie Hakim, Sheraz R. Markar, George B. Hanna

**Affiliations:** † Department of Surgery and Cancer, Imperial College London, London W12 0HS, United Kingdom; ‡ Department of Metabolism, Digestion and Reproduction, Imperial College London, London SW7 2AZ, United Kingdom; § Nuffield Department of Surgical Sciences, University of Oxford, Oxford OX3 9DU, United Kingdom

## Abstract

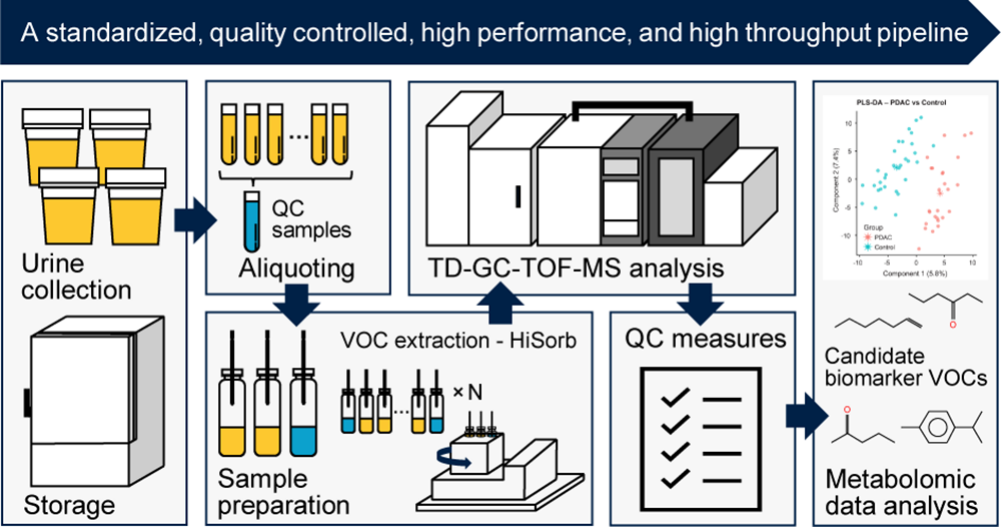

Volatolomics offers an opportunity for noninvasive detection and monitoring of human disease. While gas chromatography–mass spectrometry (GC–MS) remains the technique of choice for analyzing volatile organic compounds (VOCs), barriers to wider adoption in clinical practice still exist, including: sample preparation and introduction techniques, VOC extraction, throughput, volatolome coverage, biological interpretation, and quality control (QC). Therefore, we developed a complete pipeline for untargeted urinary volatolomic profiling. We optimized a novel extraction technique using HiSorb sorptive extraction, which exhibited high analytical performance and throughput. We achieved a broader VOC coverage by using HiSorb coupled with a set of complementary chromatographic methods and time-of-flight mass spectrometry. Furthermore, we developed a data preprocessing strategy by evaluating internal standard normalization, batch correction, and we adopted strict QC measures including removal of nonlinearly responding, irreproducible, or contaminated metabolic features, ensuring the acquisition of high-quality data. The applicability of this pipeline was evaluated in a clinical cohort consisting of pancreatic ductal adenocarcinoma (PDAC) patients (*n* = 28) and controls (*n* = 33), identifying four urinary candidate biomarkers (2-pentanone, hexanal, 3-hexanone, and *p*-cymene), which can successfully discriminate the cancer and noncancer subjects. This study presents an optimized, high-throughput, and quality-controlled pipeline for untargeted urinary volatolomic profiling. Use of the pipeline to discriminate PDAC from control subjects provides proof of principal of its clinical utility and potential for application in future biomarker discovery studies.

Volatile organic compounds (VOCs), produced within the body as a consequence of normal and aberrant metabolism, offer an opportunity for noninvasive detection and monitoring of disease states.^1−4^

While previous studies have tended to focus on the VOC signature of exhaled breath, other matrices such as urine have garnered less attention. Recent systematic reviews found promising evidence supporting urinary VOC analysis for cancer detection.^5−7^ Like exhaled breath, urine analysis is entirely noninvasive and widely accepted by patients.^6,7^ Additional advantages of urine include ease of collection and storage, VOC abundance, and the ability to generate pooled samples for quality control (QC) processes. Regarding sample preparation and introduction techniques for urinary volatolomics, the most common methods are direct headspace analysis, solid phase microextraction (SPME), and stir bar sorptive extraction (SBSE).^8^ Headspace approaches suffer from low sensitivity, while SPME and SBSE require manual handling, which can potentially introduce contamination and limit high-throughput analysis. Furthermore, the cost and fragility of SPME fibers further limit their use in large-scale clinic studies.^9^ The development of novel alternative sorptive extraction techniques, such as HiSorb sorptive extraction, offers the opportunity for broader profiling with adaptability for high-throughput analysis of clinical samples.^10^

Herein, we present a methodology for urinary volatolomics using the HiSorb. Dual polar and nonpolar column methodologies coupled with high resolution time-of-flight mass spectrometry (GC-TOF-MS) and strict QC framework are described for reliable VOC detection and identification.^11^ The performance of the optimized method was assessed using the urine of patients with pancreatic ductal adenocarcinoma (PDAC) and control patients.

## Experimental Section

### Urine Samples

A cohort of 15 healthy volunteers (8 males and 7 females, age 32 ± 5 years) was recruited for method development (REC reference 04/Q0403/119). All subjects provided informed written consent prior to participation. No special dietary restrictions were required prior to enrollment. Subjects were asked to pass urine into standard 50 mL urine specimen vials, which were immediately sealed. All samples were aliquoted into smaller 15 mL vials before being stored at −80 °C.

The methodology established using urine from healthy volunteers was subsequently evaluated in a clinical cohort consisting of 28 patients diagnosed with pancreatic ductal adenocarcinoma (PDAC) and 33 controls (REC reference 17/WA/016 and 14/LO/1136). PDAC patients and controls were recruited from Hammersmith Hospital (London, UK) between March 2016 and March 2020. Patients were recruited at the time of routine investigation or treatment of a known PDAC. Controls were recruited during routine oesophago-gastro-duodenoscopy (OGD). Inclusion criteria were as follows: (i) PDAC patients aged >18 years with biopsy confirming PDAC and (ii) controls aged >18 years presenting with or without upper gastrointestinal symptoms but with normal upper gastrointestinal tract on endoscopy. Where available, additional investigations (e.g., imaging studies) were used to verify the absence of PDAC. Patients’ medical records were also reviewed at least one year after the time of recruitment to ensure that a diagnosis of cancer had not been made during this period. Exclusion criteria were as follows: (i) patients with nonadenocarcinoma pancreatic cancers (e.g., neuroendocrine tumors), (ii) patients with benign gastrointestinal diseases, (iii) patients diagnosed with other synchronous cancers, (iv) patients with either a suspected or confirmed active infection, liver failure and/or renal failure, and finally, (v) patients who were unable to provide informed written consent or who were not able to provide a >5 mL urine sample. Patient demographics are presented in Table S1.

### Chemicals and Consumables

Analytical grade sodium chloride, methanol, hydrochloride acid (HCl), hexane, alkane standards (*n*-C_8_ to *n*-C_20_ in hexane, 40 mg/L), isotopically labeled analytical standards, including toluene-*d*_8_, acetone-*d*_6_, butyraldehyde-*d*_2_, phenol-*d*_6_, benzene-*d*_6_, and acetophenone-*d*_8_, and laser cryo-tags were purchased from Sigma-Aldrich (Gillingham, UK). Deionized nanopure water was produced by Millipore Direct-Q 3 water purification system (Merck Millipore, Watford, UK). 1.5 and 2 mL cryovials with safe lock, as well as 15 and 50 mL centrifuge tubes were purchased from Scientific Laboratory Supplies, Ltd. (Nottingham, UK). Pipettes and pipette tips were purchased from Fisher Scientific UK, Ltd. (Loughborough, UK). Clear glass 20 mL headspace vials with crimp top and round bottom, caps with HiSorb septa, HiSorb septum plugs, HiSorb handle for manual probe extraction, HiSorb agitator, HiSorb probes coated with polydimethylsiloxane (PDMS), empty inert-coated thermal desorption (TD) tubes with stainless and inert-coated stainless steel DiffLok caps, Tenax TA/Carbograph stainless sorbent TD tubes, and TC-20 TD tube conditioner were supplied from Markes International (Llantrisant, UK). Urine osmolality was measured with an OsmoPRO multisample micro-osmometer from Advanced Instruments (Horsham, UK).

### VOC Extraction

Prior to extraction, urine samples were removed from the −80 °C freezer and thawed overnight at 4 °C. Pooled volunteer urine samples were generated by mixing equal amounts (10 mL) of each healthy sample. Three technical replicates were created for each test condition. A variable volume (see Optimization of Urinary VOC Extraction section below) of pooled urine or water (blank) was transferred to glass 20 mL headspace vials. Internal standards, including toluene-*d*_8_, acetone-*d*_6_, butyraldehyde-*d*_2_, phenol-*d*_6_, benzene-*d*_6_, and acetophenone-*d*_8_ (20 mg/L in MeOH-H_2_O 1:1) were spiked, and NaCl (0.4 g) were added to each sample. The vials were sealed and left for 15 min to equilibrate.

Extraction of urinary VOCs was carried out using HiSorb sorptive extraction. TD tubes with HiSorb probes loaded were preconditioned using the TC-20 TD tube conditioner at 260 °C for 180 min under N_2_ stream at 20 psi head pressure. A single HiSorb probe was inserted into the glass headspace vial through a septum, exposing the extracting phase in the urine headspace while avoiding contact with the urine or the wall of the vial. After HiSorb probe insertion, vials were placed in a HiSorb agitator, which allowed 16 glass headspace vials to be agitated at the same time. Following the extraction, the HiSorb probes were removed from the vials using the HiSorb handle and placed back into their corresponding TD tubes, which were immediately sealed using DiffLok caps.

### Optimization of Urinary VOC Extraction

Urinary extraction conditions that were evaluated included the following: (i) extraction temperature and time; (ii) sample acidification; (iii) sample volume; (iv) sample dilution; and (v) headspace versus immersive analysis. Baseline conditions were as follows: 2 mL, undiluted, unacidified urine samples extracted at 37 °C/30 min with headspace analysis.

#### Extraction Temperature and Time

VOC extraction from a standard volume of urine (2 mL) was assessed under the following conditions: 37 °C/30 min, 37 °C/120 min, 60 °C/30 min, and 60 °C/120 min.

#### Sample Acidification

HCl (5 M) was slowly added to urine samples (5 mL) at room temperature monitored by a pH meter, until a pH of 2.0 was reached. Unacidified urine samples (5 mL) served as a control.

#### Sample Volume

Urine samples of different volumes (1, 2, 3, and 5 mL) were assessed.

#### Sample Dilution

Undiluted urine samples (2 mL) were compared to urine samples that were diluted at a ratio of 1:1 (2 mL of urine:2 mL of deionized nanopure water), 1:2 (2 mL of urine:4 mL of water), 1:3 (2 mL of urine:6 mL of water), and 1:4 (2 mL of urine:8 mL of water).

#### Headspace Versus Immersive Analysis

VOCs in a mixture of VOC standards spiked in water and VOCs in urine were extracted using both headspace and immersive analyses.

Method performance was evaluated based on comparisons of the total peak area/chemical class of 14 selected VOCs, which belong to potential cancer biomarker chemical classes,^6^ including alcohols (1-butanol), ketones (acetone, 2-butanone, 2-pentanone, and 4-heptanone), aldehydes (butanal, pentanal, hexanal, heptanal, octanal, and nonanal), and heterocyclic (furan, 2-methyl) and organosulfur compounds (dimethyl sulfide and dimethyl disulphide). For experiments comparing headspace versus immersive sampling, pooled healthy volunteer urine, standard VOC mixture, and blanks (nanopure water) were analyzed in five replicates per condition. Blank levels were subtracted and average peak areas of headspace versus immersive analysis were compared (Figure S1). All comparisons between test conditions were performed by unpaired *t*-test, with statistical significance assigned to *p* values of <0.05.

### Instrumentation

The principal analytical platform used for VOC analysis was a TOF-based Markes TD100XR thermal desorber coupled with an Agilent 7890B GC and a Markes BenchTOF select MS (Markes International, Llantrisant, UK). A Markes TD100 thermal desorber (Markes International, Llantrisant, UK) coupled with an Agilent 7890B GC and a quadrupole 5977A MS system served as a reference assay. Chromatographic separation was performed for the polar TOF assay using a Mega WAX-HT, (20 m × 0.18 mm × 0.18 μm, MEGA S.r.l., Legnano, Italy), for the nonpolar TOF assay using a DB5-MS UI (30 m × 0.25 mm × 1.00 μm, Agilent Technologies, Santa Clara, USA) and for the reference quadrupole MS assay using a Zebron ZB-624UI, 60 m × 0.25 mm × 1.40 μm (Phenomenex, Torrance, USA) capillary column.

### TD-GC-MS Settings

TD tubes with HiSorb probes loaded were initially prepurged for 1 min with He flow at 50 mL/min. Primary desorption was performed at 250 °C for 5 min to desorb the VOCs onto a cold trap (material emissions, Markes International, Llantrisant, UK) at 25 °C in split mode (1:10). Trap (secondary) desorption was performed at 250 °C (ballistic heating at 60 °C/s) for 3 min, with the flow path onto GC heated constantly at 200 °C. For sample recollection, conditioned Tenax/Carbograph-5 TD tubes were used (Markes International, Llantrisant, UK).

For the optimized TOF-based polar assay, the He flow was set at 0.7 mL/min. The oven temperature was initially set at 35 °C for 2 min and was increased to 240 °C (4 °C/min with 2 min hold). For the optimized TOF-based nonpolar assay, the He flow was set at 2 mL/min. The oven temperature was initially set at 35 °C for 4.5 min and was increased to 300 °C (10 °C/min with 4 min hold). The MS transfer line was maintained at 260 °C, and the ion source (70 eV electron impact) was at 260 °C. The MS analyzer was set to acquire over the range of 30 to 600 *m*/*z*, with the data acquisition rate at 6 Hz.

For the reference quadrupole-based method, the column flow was set at 1.0 mL/min of He. The oven temperature was initially set at 40 °C for 4 min, increased to 150 °C (15 °C/min without hold), and finally increased to 240 °C (10 °C/min with 15 min hold). The MS transfer line was maintained at 230 °C; the ion source (70 eV electron impact) was at 240 °C, and the MS quadrupole was held at 150 °C. The quadrupole was set to acquire over the range of 35 to 250 *m*/*z*, with the data acquisition rate at 6 Hz.

### Sample Recollection

Using pooled urine samples and the optimized nonpolar GC-TOF-MS method developed in the preceding section, sample recollection was evaluated. During recollection, the split flow (90% of the sample) was transferred on to a preconditioned sorbent TD tube. Using the same GC-TOF-MS method, this TD tube was analyzed and recollected a further four times.

### Urine Density Correction

Urine samples from 11 healthy volunteers (undiluted and diluted 1:1 with nanopure water) were extracted and analyzed using the optimized nonpolar GC-TOF-MS method. VOC profiles were compared with supervised multivariate statistical analysis (orthogonal projections to latent structures–discriminant analysis, OPLS-DA) before and after osmolality correction in SIMCA 15 (Sartorius, Malmö, Sweden).

### Quality Control

Pooled urine QC samples were generated for both the method development (healthy volunteers) and clinical (PDAC patients and controls) cohorts by mixing an equal amount (0.8 mL) of each study sample. For method development and the comparison of different experimental conditions, healthy volunteer pooled QC urine and blanks (deionized nanopure water) were used. For the PDAC cohort samples, one blank sample and six pooled QC samples were analyzed with every 25 clinical samples, resulting in an analytical batch of 32 samples (Table 1). Furthermore, a dilution series (0.625, 1.25, 2.5, 5, 10, 25, 50, and 75% of QC) was analyzed prior to the analysis of clinical batches.^11^ Metabolic features with a coefficient of variation (CV) of <30% were considered reproducible, and features with a 1-tailed Spearman’s rho of >0.7 and a *q* value of <0.05 after Benjamini–Hochberg correction^12,13^ were considered linear. VOC features with blank average levels of <30% in nanopure water compared to their corresponding levels in the pooled QC samples were considered to be uncontaminated. Siloxanes (artifacts generated either from chromatographic columns or extraction sorbents), features with a signal to noise ratio (S/N) of <3, or annotated features whose reverse matched factor (RMF) < 800 versus NIST 17 Mass Spectral and Retention Index Libraries (NIST, Gaithersburg, USA) penalized with retention index (RI) were removed after peak integration from downstream analysis.^14^

**Table 1:** Run Order in a Typical Analytical Batch^a^

no. of run	1	2	3–7	8	9–13	14	15–19	20	21–25	26	27–31	32
sample	B	QC1	S1–5	QC2	S6–10	QC3	S11–15	QC4	S16–20	QC5	S21–25	QC6

a B: blank; QC: pooled quality control sample; S: study sample.

### Data Extraction, Preprocessing, and Statistical Analysis

Data acquisition was performed with ChromSpace (Markes International, Llantrisant, UK) and quadrupole data with MassHunter (Agilent Technologies, Santa Clara, USA). ChromSpace followed by Gavin^15^ was used for deconvolution, peak picking, and integration. All data files were dynamically baseline corrected (DBC) in ChromSpace before further analysis. Mass spectra with their corresponding RI were searched in NIST 17. High-quality data were acquired by deleting nonlinear, irreproducible, or contaminated metabolic features, performing best-matched internal standard normalization^16^ and performing QC normalization, which can correct interbatch analytical variabilities.^11^ Details of data normalization are presented in the Supporting Information. Univariate statistical analyses were performed with SPSS 25 (SPSS Inc., Chicago, USA) and MATLAB R2022a (MathWorks, Natick, USA). Figures were generated with Prism 9 (GraphPad Software Inc., La Jolla, USA). Multivariate statistical analysis was performed on R 4.1.2 (R Foundation for Statistical Computing, Vienna, Austria). Total area normalization was applied to each sample. Principal component analysis (PCA) was used to investigate the structure of the data and remove possible outliers using the “mixOmics” package.^17^ Polar and nonpolar datasets were then combined before generating partial least square–discriminant analysis (PLS-DA) models. PLS-DA model performance was evaluated using leave-one-out (loo) cross-validation and models with a classification error rate (CER) of <0.5 were considered informative. Metabolites with a variable importance projection score (VIP) of >1.5 were considered relevant for group separation. Loadings were extracted from the different models to identify group contribution. Four endogenous metabolites with the highest VIP scores were identified versus authentic standards (Figure S4) and used for the generation of biomarker-specific PLS-DA models. The dataset was split into training and test data (3/4 and 1/4 of the data, respectively) using the “tidymodels” package before evaluating model performance.^18^ The “auroc” function from the mixOmics package was used to generate the final receiver operating characteristic (ROC) curve on the test data. Additionally, ROC curves for each biomarker were generated from the full dataset using MetaboAnalyst 5.0 (Xia Lab @ McGill, Ste. Anne de Bellevue, Canada).^19^ The code used for multivariate data analysis is available on GitHub (https://github.com/simonezuffa/Manuscript_HiSorb).

## Results and Discussion

### VOC Extraction

**Figure 1:**
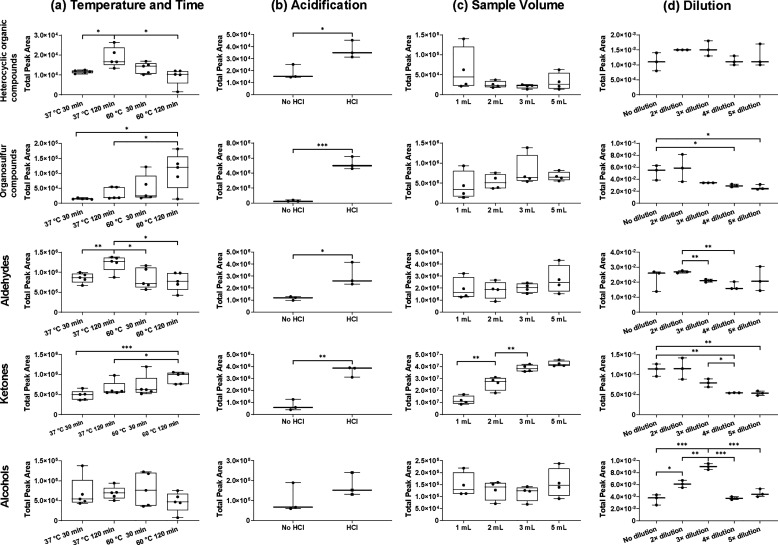
Optimization of HiSorb extraction conditions. Effects of (a) extraction temperature and time, (b) acidification, (c) sample volume, and (d) dilution were evaluated. The total peak area of 14 selected VOCs, which belong to five potential cancer biomarker chemical classes, including alcohols (1-butanol), ketones (acetone, 2-butanone, 2-pentanone, and 4-heptanone), aldehydes (butanal, pentanal, hexanal, heptanal, octanal, and nonanal), heterocyclic (furan, 2-methyl) and organosulfur compounds (dimethyl sulfide and dimethyl disulphide), was compared. Unpaired *t*-test was used for condition-by-condition comparison. Boxplots represent lower, upper quartile, and interquartile range (IQR); whiskers represent minimum and maximum values; *: *p* < 0.05, **: *p* < 0.01, ***: *p* < 0.001.

A summary of the findings of VOC extraction optimization experiments is presented in Figure 1. With the exception of heterocyclic organic compound and aldehydes (37 °C/120 min) and organosulfur compounds and ketones (60 °C/120 min), there was no statistically significant difference among the four temperature and time conditions (Figure 1a). This indicated that for most compounds, extraction temperature and time did not have a significant impact on VOC detection when using the chosen HiSorb extraction method. Therefore, in the interest of time efficiency, 30 min is proposed to be a suitable extraction time for future clinical trials. In addition, extracting by HiSorb probes at 60 °C provided higher total peak area in most chemical groups.

For all chemical groups, samples acidified with HCl had significantly higher total peak area when compared to unacidified samples (Figure 1b). This finding suggests that adding acid to urine samples increases total peak area and should therefore be carried out routinely. This is supported by other studies, which showed that higher numbers and concentration of urinary VOCs are detected in an acid environment compared to a neutral or alkaline environment.^20−22^ Although there are limited examples within the literature, HCl is the most common acid used for urine acidification.^21^ Changing the pH can alter the urine sample matrix by increasing the decomposition and degradation of selected compounds, especially when a powerful oxidizing acid is used. It may also cause more compounds to transition from liquid to gas phase by increasing activity coefficients and decreasing partition coefficients.^21^ Therefore, testing pH adjustment effects before adopting it in the volatile profiles is recommended.

For the majority of chemical classes, sample volume did not appear to have a significant effect on total peak area (Figure 1c). For 1 mL samples, higher variability was however observed, raising doubts about extraction stability. Thus, a minimum sample volume of 2 mL is considered adequate for urinary VOC analysis to achieve optimal extraction efficiency.

Urine is considered to be a rich source of VOCs. In such circumstances, the large number of components may negatively impact on analysis of trace compounds, establishing a matrix effect.^23^ The dilution of urine samples has the potential to compensate for such matrix effects. In the current study, the total peak area of all chemical groups except alcohols for undiluted samples were higher than diluted ones, and the variation of undiluted samples were often higher (Figure 1d). As a result, sample dilution is not recommended to ensure optimal extraction efficiency.

Based on the above observations, the selected HiSorb extraction conditions were: 2 mL of undiluted and acidified urine at 60 °C for 30 min. These conditions appear to offer both reliable VOC detections with the opportunity for high-throughput analysis that is desired within clinical trials.

### GC–MS Optimization

Two GC-TOF-MS methods were evaluated, one with a thin phase polar column designed for separation of acidic VOCs and a second with a general-purpose thick phase nonpolar column that provided coverage for remaining VOC classes.

The performance of the four polar column assays is shown in Table 2. The highest number of urinary VOCs (*n* = 121) (Table S2) that were linear, reproducible, considered noncontaminant, and library matched was observed with 2 mL of sample volume, 0.7 mL/min column flow (close to optimum linear velocity), and a slow temperature gradient (57 min). Remarkably, for lower sample volume (0.25 mL), greater performance was observed with the high flow rate method where in just 17 min, 85 VOCs were identified. This can be explained by the highly retentive nature of the WAX columns, which in long gradients tend to generate narrow peaks with a lower signal to noise ratio.

**Table 2:** Four-Assay Panel of GC Methods (Polar Column)

instrument	TD-GC-TOF-MS (polar column)			
urine volume	2 mL	0.25 mL	2 mL	0.25 mL
GC column flow	0.7 mL/min	0.7 mL/min	1.2 mL/min	1.2 mL/min
GC oven gradients	initial temperature at 35 °C hold for 2 min, ramp to 240 °C at 4 °C/min hold for 2 min		initial temperature at 35 °C hold for 1.9 min, ramp to 240 °C at 20 °C/min hold for 0.2 min	
GC cycle time	57 min	57 min	17 min	17 min
no. of VOCs	121	33	96	85

For the nonpolar assays, a longer column with a thicker phase was chosen to achieve a broad VOC coverage. The highest VOC yield (*n* = 167) (Table S3) was observed with 2 mL of sample volume, 2 mL/min column flow, and a faster temperature gradient (37 min) (Table 3).

**Table 3:** Four-Assay Panel of GC Methods (Nonpolar Column)

instrument	TD-GC-TOF-MS (nonpolar column)			
urine volume	2 mL	0.25 mL	2 mL	0.25 mL
GC column flow	1.6 mL/min	1.6 mL/min	2 mL/min	2 mL/min
GC oven gradients	initial temperature at 35 °C hold for 4.5 min, ramp to 300 °C at 8 °C/min hold for 4 min		initial temperature at 35 °C hold for 4.5 min, ramp to 300 °C at 10 °C/min hold for 4 min	
GC cycle time	44 min	44 min	37 min	37 min
no. of VOCs	154	85	167	103

The overlap between “reliable” urinary VOCs detected by the highest performing polar and nonpolar methods was less than 20% (*n* = 23) (Tables S2 and S3). While some annotations may be inaccurate due to the nature of untargeted analysis, it is evident that the two methods are highly complementary. Furthermore, it must be noted that this was observed even with the nonpolar PDMS sorbent, which prioritizes the absorption of the less polar VOCs. Thus, in a potential multibed sorbent approach, the complementarity of these methods would be expected to be even greater. In total, 455 urinary VOCs were observed using these methodologies. Forty-one (9% of the total) of these compounds have previously been detected in human urine, according to a recent review by Drabińska et al.^5^ Importantly, unlike in previous studies, VOCs reported by this study were also subject to QC filtering measures.^5^ The methodology proposed therein therefore offers both a deep and high-quality profile of the human urinary volatolome, highlighting its potential applicability in future biomarker discovery studies.

### Sample Recollection

Results of sample recollection experiments are presented in Table 4. After one recollection cycle, 147/167 (94.6%) VOCs were retained, falling to 133 (79.6%) VOCs by the fourth recollection. Accordingly, in clinical studies, where sample volumes maybe limited, sample recollection enables the option for multiplatform analyses of a single sample.

**Table 4:** Performance of Sample Recollection

cycle	original run	recollection 1	recollection 2	recollection 3	recollection 4
instrument	TD-GC-TOF-MS (nonpolar column)				
urine volume	2 mL				
no. of VOCs	167	158	147	147	133
% of VOC recovered	100%	94.6%	88.0%	88.0%	79.6%

### Urine Density Correction

Urine concentration is regulated by renal function and is subject to wide variation both in healthy and disease states. Under normal physiological conditions, urine volume has been found to fluctuate by up to 15-fold, resulting in a significant variation in urinary metabolite levels.^24^ Therefore, in order to improve data quality, several strategies have been investigated.^25^ To account for this effect, creatinine adjustment is the most widely applied, but its use is controversial since creatinine levels are affected by muscle mass and coexisting disease states. Thus, osmolality correction, which accounts for the number of dissolved particles per unit of water in urine,^24^ was investigated in the presented volatolomic assays, as a more independent method of accounting for urine dilution. Osmolality normalization did not appear to improve data quality and, counterintuitively, no correlation between sample total peak areas and osmolality was observed (Figure S2).

### Application in PDAC Patients and Controls

Urine samples from 61 patients, 15 pooled QC samples, and 3 blanks were analyzed in three batches. Urine (2 mL) was profiled both with the optimal 57 min polar and the optimal 37 min nonpolar assay (see Tables 2 and 3). Internal standard normalization and QC normalization yielded the highest number of reliable urinary VOC features (Supporting Information and Table S4). Therefore, they were adopted for data preprocessing.

**Figure 2:**
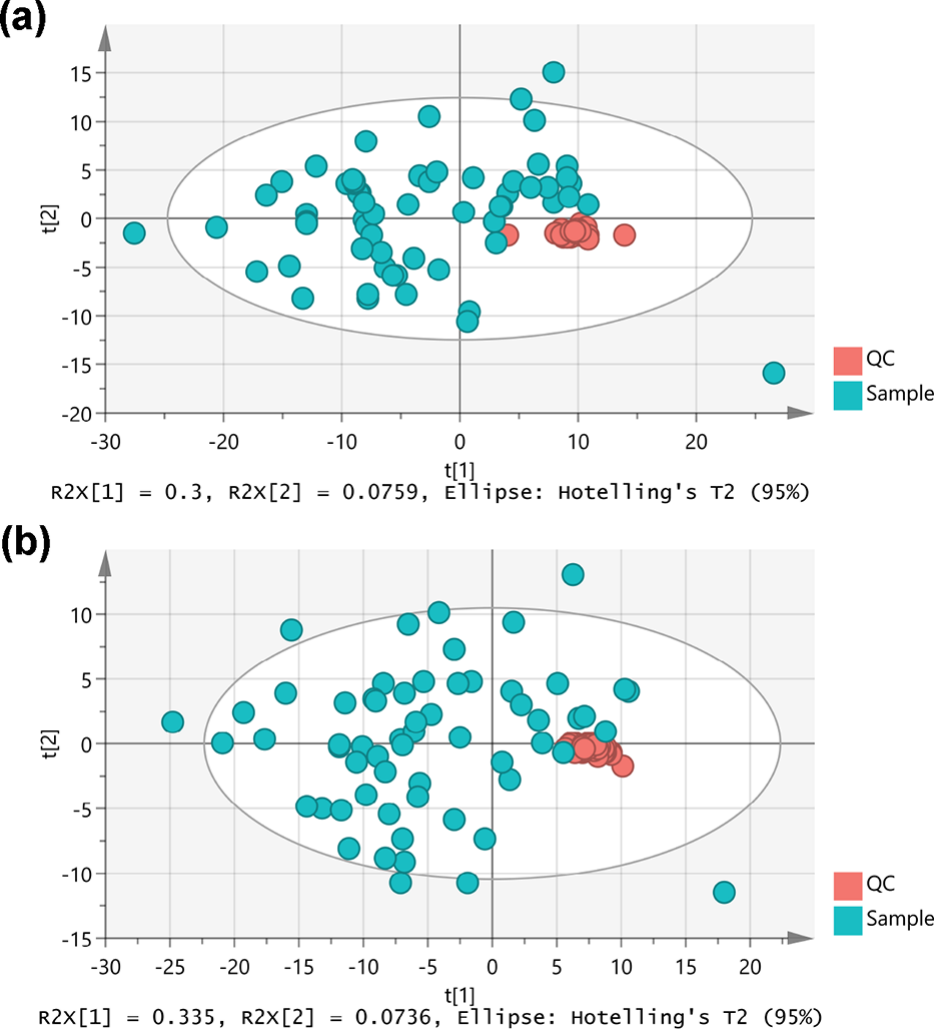
PCA score plots for (a) polar and (b) nonpolar datasets. QC data points clustered tightly, in comparison to the total variance in the projection, indicating high data quality.

Extracted and preprocessed metabolic profiles, both polar and nonpolar, obtained with Gavin were assessed with PCA. PCA plots revealed a tight cluster of QC samples that were surrounded by study samples (Figure 2), suggesting high data quality.^11^

**Figure 3:**
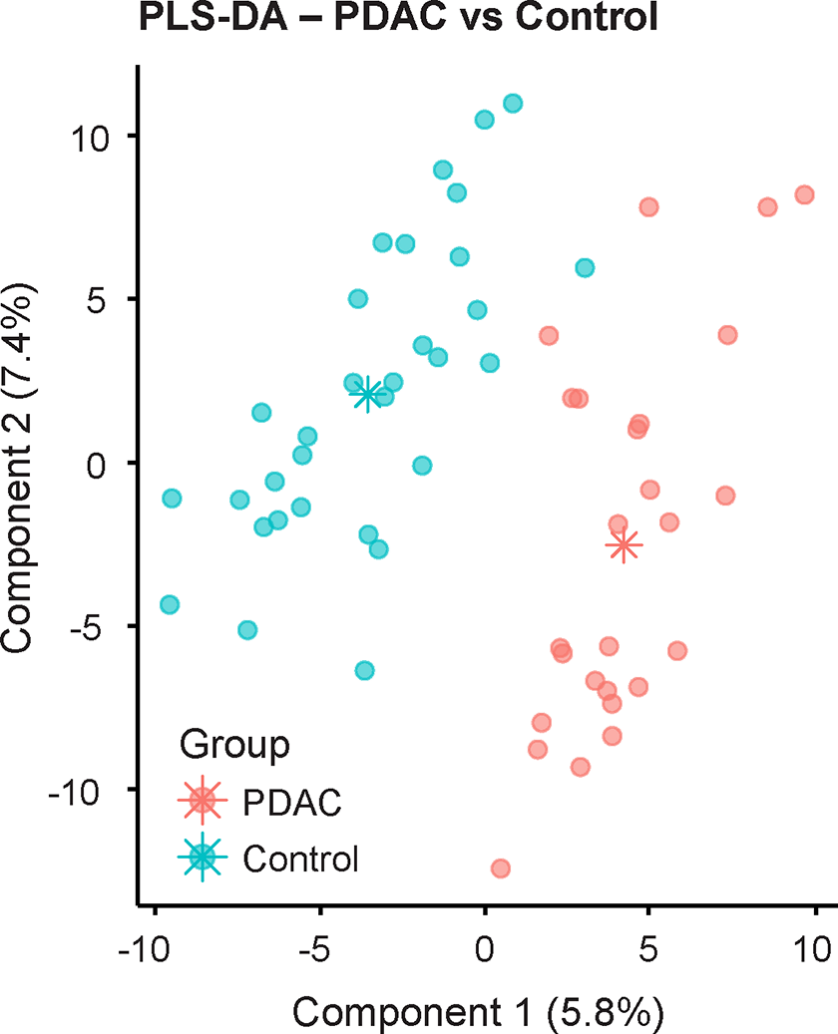
Score plot of the PLS-DA model generated on the whole dataset, combining polar and nonpolar datasets. The classification error rate (CER) of the model was 0.23, indicating informative classification.

Polar and nonpolar datasets were then combined to generate a single PLS-DA model (CER 0.23) (Figure 3). Seventy metabolites had a VIP score of >1.5 (Table S5) and of these, 9 were present in higher abundance in PDAC patients, while 61 were higher in controls. The top 25 most discriminant and annotated metabolites were then further investigated. Four endogenous metabolites (2-pentanone, hexanal, 3-hexanone, and *p*-cymene, Table 5) were then retained and used to rebuild a final PLS-DA model (CER 0.18), for which an ROC curve with area under the curve (AUC) 0.82 (p value 0.037) was generated (Figure S3). ROC curves for each individual target metabolites are also presented in Figure S3. These findings demonstrated that using the proposed analytical pipeline may successfully differentiate PDAC patients from control subjects based on four urinary VOCs.

**Table 5:** Candidate Biomarker VOCs for PDAC Diagnosis

top ions	compound name	CAS no.	chemical class	increase/decrease in PDAC
43, 86, 41	2-pentanone	107-87-9	ketone	↓
44, 56, 41	hexanal	66-25-1	aldehyde	↓
43, 57, 71	3-hexanone	589-38-8	ketone	↓
119, 134, 91	*p*-cymene	99-87-6	aromatic	↓

Due to a paucity of mechanistic studies focusing on human VOC metabolism,^6^ the biological significance of the identified metabolites remains largely unclear. Only one of the identified VOCs, 2-pentanone, has previously been linked to pancreatic cancer in the literature. Daulton et al. reported that urinary 2-pentanone is able to separate urine of PDAC versus chronic pancreatitis (CP) patients and CP versus healthy subjects, with ROC-AUC 0.75.^26^ 2-pentanone has also been found to be associated with ulcerative colitis,^27^ celiac disease,^28^ nonalcoholic fatty liver disease,^29^ and Crohn’s disease,^27^ suggesting that it may serve as a more general biomarker of inflammation.

Hexanal has previously been reported as urinary biomarker of prostate,^30^ bladder,^31^ and lung cancer.^32^ As a short chain aldehyde, it can be produced by peroxidation of unsaturated fatty acids in many parts of the body^33^ and also by oxidation of 2,2,6-trimethyl-cyclohexanone and 3-hexanone.^5^

Previous studies have also linked urinary 3-hexanone with lung, breast, and colon cancer^34^ and *p*-cymene with colorectal cancer, lymphoma, leukemia, and breast cancer.^35,36^ Further studies are now needed to both independently validate those biomarkers in a larger patient cohort and to explore the underlying biology of their production in PDAC.

The optimized urinary VOC analytical pipeline worked in a high-throughput, automated, and reliable manner, allowing for 25 clinical samples to be analyzed in a single day. This analysis capability could be increased on demand if an adequate supply of HiSorb probes and GC–MS instruments is available, highlighting its potential for large-scale clinical adoption.

The use of HiSorb probes coated with PDMS that extract mainly nonpolar compounds was an acknowledged limitation of this study. It is anticipated that the ability to explore the contribution of polar compounds to PDAC detection would further improve model performance. A multibed HiSorb probe will aid in the detection of VOCs ranging from polar to nonpolar. A further limitation of this study was that observations were made from a relatively small number of healthy subjects and patients both in terms of method development and clinical application stages. A follow-up study enrolling the appropriate number of patients is needed, which will enable the conducting of a cohort for VOC biomarker discovery and an independent validation cohort to verify the results.

## Conclusions

The study has presented an optimized and quality-controlled pipeline for untargeted urinary volatolomic profiling with sorptive extraction and GC-TOF-MS. The clinical utility of this pipeline was demonstrated by its ability to differentiate the urinary volatile profiles of PDAC patients and controls.

Findings underly a potential future role for urinary VOCs as a noninvasive method for disease detection and monitoring. Inclusion of QC measures within a standardized pipeline offers the opportunity for analytical reliability with multicenter trials and support wider clinical adoption.

## Supporting Information Available

Supplemental figures and tables detailing study cohort characteristics, method development, evaluation of osmolality normalization, identified urinary VOCs, data preprocessing, PDAC related metabolites, and ROC curves (PDF)
