# Supplementary material for: A Complete Pipeline for Untargeted Urinary Volatolomic Profiling with Sorptive Extraction and Dual Polar and Nonpolar Column Methodologies Coupled with Gas Chromatography Time-of-Flight Mass Spectrometry
Source: Anal Chem. 2023 Jan 5;95(2):758–65. doi: 10.1021/acs.analchem.2c02873 (PMC9850407; doi:10.1021/acs.analchem.2c02873)
Supplement: Supplementary file 1 — ac2c02873_si_001.pdf [file ac2c02873_si_001.pdf]

Supporting Information

A Complete Pipeline for Untargeted Urinary Volatolomic Profiling with Sorptive Extraction and GC-TOF-MS

Qing Wen[1§], Antonis Myridakis[1§], Piers R. Boshier[1], Simone Zuffa[2], Ilaria Belluomo[1], Aaron G. Parker[1], Sung-Tong Chin[1], Stephanie Hakim[1], Sheraz R. Markar[1, 3], and George B. Hanna[1]*

[1] Department of Surgery and Cancer, Imperial College London, London W12 0HS, United Kingdom

[2] Department of Metabolism, Digestion and Reproduction, Imperial College London, London SW7 2AZ, United Kingdom

[3] Nuffield Department of Surgical Sciences, University of Oxford, Oxford, OX3 9DU, United Kingdom

* Email: g.hanna@imperial.ac.uk

[§] Q.W. and A.M. contributed equally to this paper.

TABLE OF CONTENTS

Figure S1. Evaluation of headspace analysis versus immersive analysis. S2

Figure S2. Evaluation of osmolality normalization for urine density correction. S3

Figure S3. ROC curves for PDAC versus controls. S4

Figure S4. Candidate biomarker confirmation with authentic standards. S5

Table S1. Characteristics of the study cohort. S6

Table S2. List of VOCs from urine of healthy individuals identified by the optimal polar GC-TOF assay. S7

Table S3. List of VOCs from urine of healthy individuals identified by the optimal non-polar GC-TOF assay. S10

Details on data pre-processing for the application in PDAC patients and controls. S15

Table S4. Number of reliably measured VOCs after data pre-processing and feature filtering. S15

Table S5. List of urinary VOCs with variable importance projection score (VIP) > 1.5. S16

References S18

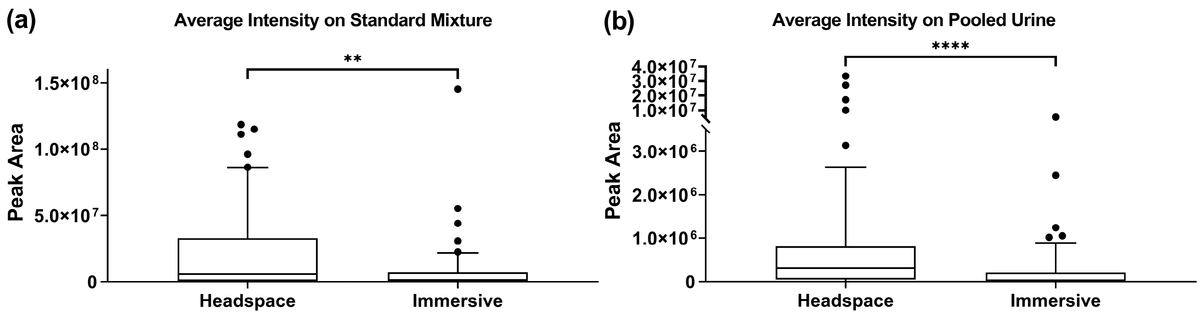

Figure S1. Evaluation of headspace analysis versus immersive analysis.

(a) Average peak area of VOCs in a mixture of VOC standards spiked in water. (b) Average peak area of VOCs in urine. Mann-Whitney U test was used for comparison. Boxplots represent lower, upper quartile, and interquartile range (IQR); whiskers represent 10-90 percentile; *: $p < 0.05$, **: $p < 0.01$, ***: $p < 0.001$, ****: $p < 0.0001$.

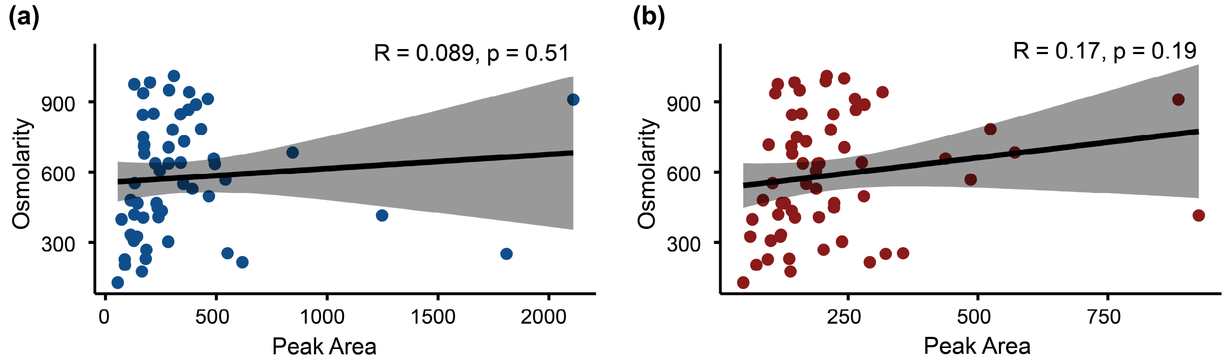

Figure S2. Evaluation of osmolality normalization for urine density correction.

(a) Polar dataset, (b) non-polar dataset. No correlation between sample peak areas and osmolality was observed using Pearson correlation.

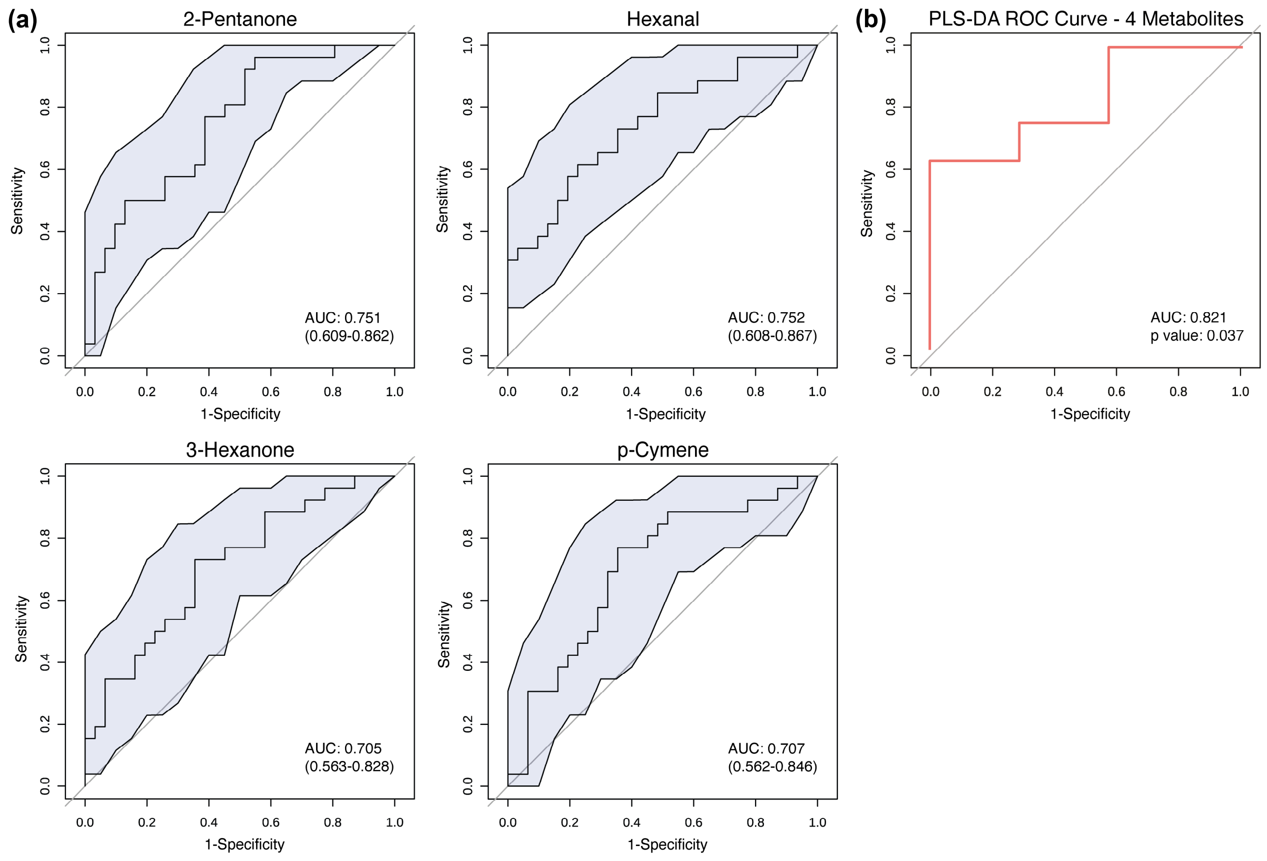

Figure S3. ROC curves for PDAC versus controls.

ROC curves were built (a) for each of the four endogenous metabolites with highest variable importance projection scores (VIPs), including 2-pentanone, hexanal, 3-hexanone, and p-cymene; and (b) for all of them combined.

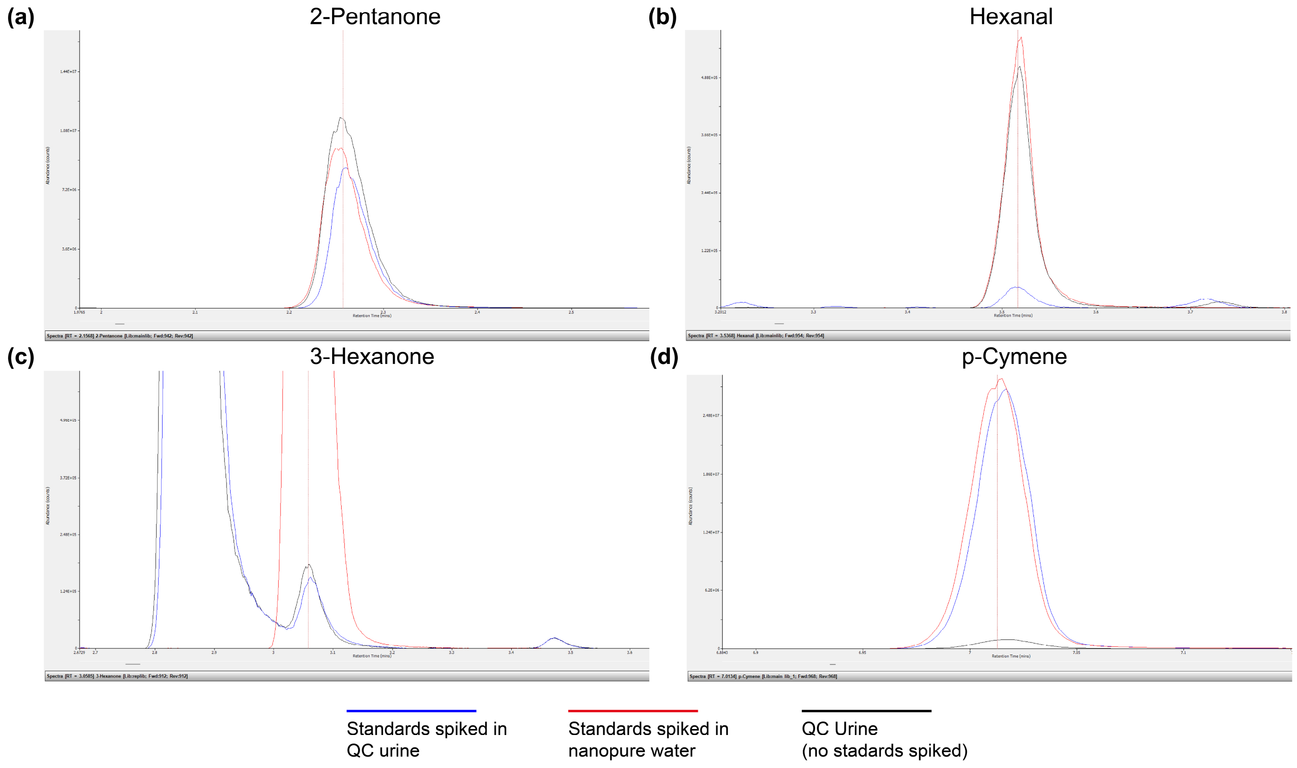

Figure S4. Candidate biomarker confirmation with authentic standards.

Original standards including (a) 2-pentanone, (b) hexanal, (c) 3-hexanone, and (d) p-cymene were analyzed in: Blue: spiked in pooled urine; Red: in standard solutions, and; Black: compared them with the native peaks of not-spiked urine samples.

Table S1. Characteristics of the study cohort.

	Pancreatic ductal adenocarcinoma (PDAC) (*n* = 28)	Control (*n* = 33)	p value
Demographics			
Age (Mean ± SD)	67.9 ± 12.2	52.7 ± 17.9	0.571
Gender (M : F)	18 : 10	18 : 15	0.441
Primary tumour (T)			
T1	0 (0%)	-	
T2	0 (0%)	-	
T3	12 (43%)	-	
T4	12 (43%)	-	
Unknown	4 (14%)	-	
Reginal lymph node (N)			
N0	5 (18%)	-	
N1	16 (57%)	-	
N2	3 (11%)	-	
Unknown	4 (14%)	-	
Distant metastasis (M)			
M0	9 (32%)	-	
M1	9 (32%)	-	
Mx	6 (22%)	-	
Unknown	4 (14%)	-	
Chemotherapy	23 (82%)	0 (0%)	1
Antibiotics	4 (14%)	3 (9%)	0.526
Diabetes	13 (46%)	3 (9%)	< 0.001

Comparisons were performed with Pearson Chi-Squared test.

Table S2. List of VOCs from urine of healthy individuals identified by the optimal polar GC-TOF assay.

No.	RT	Top Ions	RI	Compound Name	CAS No./<br>NIST No.
1	1.451	41, 72, 39	459	1-Octanamine	111-86-4
2	1.460	43, 58, 32	462	Acetone	67-64-1
3	1.495	74, 59, 43	473	Urea, N-ethyl-N-nitroso-	759-73-9
4	1.635	82, 53, 81	518	Furan, 3-methyl-	930-27-8
5	2.107	79, 77, 94	667	1,4-Cyclohexadiene, 1-methyl-	4313-57-9
6	2.126	96, 95, 43	673	Furan, 2,5-dimethyl-	625-86-5
7	2.163	85, 41, 55	685	1-Methoxy-3-methyl-2-butene	22093-99-8
8	2.243	96, 67, 95	710	2,4-Dimethylfuran	3710-43-8
9	2.271	91, 107, 79	719	Bicyclo[3.1.1]hept-2-en-6-one, 2,7,7-trimethyl-	473-06-3
10	2.352	86, 32, 43	745	2-Pentanone	107-87-9
11	2.577	71, 43, 114	816	Hexane, 3,3,4,4-tetramethyl-	5171-84-6
12	2.942	95, 32, 110	917	2-Furoic acid, pentafluorophenyl ester	355170
13	3.249	43, 57, 71	968	3-Hexanone	589-38-8
14	3.262	71, 43, 41	971	3-Buten-2-ol, 2-methyl-	115-18-4
15	3.299	43, 109, 110	977	Furan, 2,3,5-trimethyl-	10504-04-8
16	3.488	94, 45, 79	1005	Disulfide, dimethyl	624-92-0
17	3.605	57, 41, 39	1015	di-tert-Butyl dicarbonate	24424-99-5
18	3.737	44, 41, 56	1027	Hexanal	66-25-1
19	4.102	43, 41, 42	1059	1-Propanol, 2-methyl-	78-83-1
20	4.107	109, 41, 57	1060	2-Cyclopenten-1-one, 3,5,5-trimethyl-	24156-95-4
21	4.130	124, 109, 95	1062	1-Propanone, 1-(2-furanyl)-	3194-15-8
22	4.236	68, 71, 139	1071	2H-Pyran, 2-ethenyltetrahydro-2,6,6-trimethyl-	7392-19-0
23	4.362	59, 45, 87	1083	2-Pentanol, 2-methyl-	590-36-3
24	4.555	43, 71, 41	1100	3-Hexanone, 2-methyl-	7379-12-6
25	4.595	43, 57, 55	1102	1-Pentanol, 2,2-dimethyl-	2370-12-9
26	4.721	45, 207, 103	1109	Aminocaproic acid	60-32-2
27	4.907	121, 93, 79	1120	2-Carene	554-61-0
28	5.196	57, 41, 56	1136	Butane, 1,1,1-[methylidynetris(oxy)]tris-	588-43-2
29	5.623	91, 111, 106	1160	7-Oxabicyclo[2.2.1]heptane, 1-methyl-4-(1-methylethyl)-	470-67-7
30	5.833	58, 43, 71	1172	2-Heptanone	110-43-0
31	5.878	109, 79, 94	1174	2,3-Dehydro-1,8-cineole	92760-25-3
32	6.205	93, 77, 71	1193	β-Phellandrene	555-10-2
33	6.215	71, 43, 41	1193	2-(1-Adamantyl)piperidine	195243-62-0
34	6.348	69, 42, 98	1201	(R)-(+)-3-Methylcyclopentanone	6672-30-6
35	6.389	138, 95, 98	1202	Cyclohexene, 1-methyl-3-(1-methylethyl)-	13828-31-4
36	6.469	43, 58, 91	1206	2-Hexanone, 4-methyl-	105-42-0
37	6.780	55, 42, 41	1220	1-Butanol, 3-methyl-	123-51-3
38	6.894	109, 138, 79	1225	1-Propanone, 1-(5-methyl-2-furanyl)-	10599-69-6
39	7.073	81, 82, 53	1233	Furan, 2-pentyl-	3777-69-3
40	7.127	111, 138, 126	1235	Pyrimido[5,4-d]pyrimidine, 2,4,6,8-tetrakis(m-chloroanilino)-	18710-95-7
41	7.244	93, 91, 119	1241	γ-Terpinene	99-85-4

42	7.260	91, 39, 134	1241	p-Mentha-1,5,8-triene	21195-59-5
43	7.293	81, 53, 112	1243	Furan, 2-(methoxymethyl)-	13679-46-4
44	7.453	122, 43, 107	1250	Phenol, 2,4-dimethyl-	105-67-9
45	7.704	57, 43, 72	1261	3-Octanone	106-68-3
46	7.794	68, 56, 31	1265	Acetic acid, 2-propylphenyl ester	35922-83-9
47	7.894	119, 91, 134	1270	p-Cymene	99-87-6
48	7.900	42, 55, 41	1270	1-Pentanol	71-41-0
49	8.087	59, 56, 60	1278	Propylamine	107-10-8
50	8.209	93, 121, 136	1284	Cyclohexene, 1-methyl-4-(1-methylethylidene)-	586-62-9
51	8.561	43, 58, 122	1300	2-Octanone	111-13-7
52	9.165	43, 57, 41	1322	Methyl isobutyrate	547-63-7
53	9.473	83, 55, 56	1334	3-Ethylcyclopentanone	10264-55-8
54	10.139	59, 128, 43	1359	1,7-Octanediol, 3,7-dimethyl-	107-74-4
55	10.647	39, 99, 41	1378	Allyl Isothiocyanate	57-06-7
56	11.279	137, 57, 67	1402	cis-4a-Methyl-decahydronaphthalene	2547-26-4
57	11.434	92, 91, 43	1407	Benzyl isopentyl ether	122-73-6
58	11.571	82, 138, 54	1412	Isophorone	78-59-1
59	12.271	43, 113, 59	1436	2-Pentenoic acid, 4-oxo-, methyl ester, (Z)-	19522-27-1
60	12.703	117, 132, 91	1451	Benzene, 1-methyl-4-(1-methylethenyl)-	1195-32-0
61	12.769	159, 175, 174	1454	p-(1-Propenyl)-toluene	429549
62	13.274	59, 43, 94	1471	2-Furanmethanol, 5-ethenyltetrahydro-α,α,5-trimethyl-, cis-	5989-33-3
63	13.299	112, 69, 41	1472	Cyclohexanone, 5-methyl-2-(1-methylethyl)-, cis-	491-07-6
64	13.506	73, 69, 55	1479	3-Octanol, 3,6-dimethyl-	151-19-9
65	13.754	96, 95, 159	1488	Furfural	98-01-1
66	13.830	68, 67, 83	1491	2H-Pyran, 3,6-dihydro-4-methyl-2-(2-methyl-1-propenyl)-	1786-08-9
67	13.912	119, 134, 146	1494	Benzene, 1,2,4,5-tetramethyl-	95-93-2
68	14.074	112, 41, 69	1499	Cyclohexanone, 5-methyl-2-(1-methylethyl)-, (2R-cis)-	1196-31-2
69	14.076	59, 94, 67	1499	1-Butanol, 3-methoxy-	2517-43-3
70	14.362	138, 107, 149	1510	(3R,9aR)-3-Butyloctahydro-1H-pyrrolo[1,2-a]azepine	929631-67-4
71	14.534	59, 55, 41	1516	7-Octen-2-ol, 2,6-dimethyl-	18479-58-8
72	14.914	179, 119, 109	1530	Benzoic acid, 2-dimethylamino-5-phenylsulfonylamino-	263392
73	15.001	57, 31, 83	1533	1-Penten-3-ol	616-25-1
74	15.452	85, 186, 184	1550	2(3H)-Furanone, 5-hexyldihydro-	706-14-9
75	15.527	138, 139, 123	1552	(Z)-7-((5R,8R,8aS)-8-Methyloctahydroindolizin-5-yl)hept-3-en-2-ol	367215
76	15.695	41, 43, 55	1558	2-Nonenal, (E)-	18829-56-6
77	15.804	159, 59, 57	1562	Naphthalene, 1,2,3,4-tetrahydro-1,1,6-trimethyl-	475-03-6
78	15.973	43, 95, 138	1569	4-Acetyl-1-methylcyclohexene	6090-09-01
79	16.034	135, 109, 79	1571	N-Benzylformamide	6343-54-0
80	16.667	41, 93, 71	1594	Linalool	78-70-6
81	16.790	110, 109, 87	1598	Benzenethiol	108-98-5
82	17.067	43, 99, 55	1609	3-Pentenoic acid, 4-methyl-	504-85-8
83	17.446	67, 95, 68	1622	Cyclohexene, 1-methyl-4-(1-methylethyl)-, (R)-	1195-31-9
84	17.623	177, 133, 178	1629	1-naphthalenesulfonamide, 5-(acetyloxy)-N-[4-(diethylamino)-2-methylphenyl]-	399203

85	17.765	95, 81, 138	1634	Bicyclo[2.2.1]heptane, 1,7,7-trimethyl-	464-15-3
86	17.896	105, 77, 51	1639	Benzoic acid, methyl ester	93-58-3
87	18.348	71, 82, 67	1655	1,5,7-Octatrien-3-ol, 3,7-dimethyl-	29957-43-5
88	18.514	91, 119, 120	1661	Benzaldehyde, 4-methyl-	104-87-0
89	18.948	43, 55, 67	1677	1,5-Pentanediol, 3-methyl-	4457-71-0
90	18.974	81, 95, 71	1678	Cyclohexanol, 5-methyl-2-(1-methylethyl)-, (1α,2β,5α)-(±)-	15356-70-4
91	19.195	111, 55, 67	1686	2(3H)-Furanone, 5-ethenyldihydro-5-methyl-	1073-11-6
92	19.491	42, 45, 43	1697	Mercaptamine	60-23-1
93	19.515	91, 134, 119	1698	Ethanone, 1-(4-methylphenyl)-	122-00-9
94	20.010	146, 145, 117	1716	Benzofuran, 4,7-dimethyl-	28715-26-6
95	20.471	59, 93, 68	1733	α-Terpineol	98-55-5
96	20.604	110, 82, 109	1738	2-Cyclohexen-1-one, 3-methyl-6-(1-methylethyl)-	89-81-6
97	20.853	82, 54, 39	1748	Carvone	99-49-0
98	20.943	157, 142, 141	1751	Naphthalene, 1,2-dihydro-1,5,8-trimethyl-	4506-36-9
99	20.986	173, 188, 174	1753	Benzimidazole, 5-tert-butyl-2-methyl-	5805-62-9
100	21.398	97, 55, 69	1768	2-Thiopheneacetic acid, 1-cyclopentylethyl ester	278960
101	21.742	111, 68, 112	1781	1-[(2-Thienylcarbonyl)oxy]-2,5-pyrrolidinedione	83039-60-5
102	21.820	150, 91, 84	1784	Benzeneacetic acid, methyl ester	101-41-7
103	22.070	133, 105, 148	1793	Ethanone, 1-(2,5-dimethylphenyl)-	2142-73-6
104	22.375	42, 70, 43	1805	2H-Pyran-2-one, tetrahydro-6-methyl-	823-22-3
105	22.411	129, 128, 144	1806	Naphthalene, 1,2-dihydro-4-methyl-	4373-13-1
106	22.704	119, 91, 105	1818	(2R,8R,8aS)-8,8a-Dimethyl-2-(prop-1-en-2-yl)-1,2,3,7,8,8a-hexahydronaphthalene	5090-61-9
107	22.962	173, 174, 188	1828	1H-Indene, 2,3-dihydro-1,1,2,3,3-pentamethyl-	1203-17-4
108	23.234	69, 121, 41	1839	2-Buten-1-one, 1-(2,6,6-trimethyl-1,3-cyclohexadien-1-yl)-, (E)-	23726-93-4
109	24.109	91, 109, 119	1874	2-Cyclohexen-1-ol, 2-methyl-5-(1-methylethenyl)-, (1S-trans)-	2102-58-1
110	25.334	157, 142, 141	1923	4-Isopropyl-6-methyl-1-methylene-1,2,3,4-tetrahydronaphthalene	50277-34-4
111	25.412	145, 160, 118	1926	Benzene, 4-(2-butenyl)-1,2-dimethyl-, (E)-	54340-86-2
112	26.904	122, 123, 206	1986	Lauric acid, 3,5-dimethylphenyl ester	357926
113	27.741	43, 175, 157	2021	4-(2,6,6-Trimethylcyclohexa-1,3-dienyl)but-3-en-2-one	1203-08-3
114	28.449	94, 66, 65	2052	Phenol	108-95-2
115	28.860	185, 200, 157	2070	α-Corocalene	20129-39-9
116	28.892	164, 149, 77	2072	Benzene, 4-ethenyl-1,2-dimethoxy-	6380-23-0
117	29.019	119, 105, 134	2077	Spiro[3.5]nona-5,7-dien-1-one, 5,9,9-trimethyl-	91531-36-1
118	30.205	107, 108, 77	2129	p-Cresol	106-44-5
119	30.811	184, 169, 128	2155	Chamazulene	529-05-5
120	31.093	155, 41, 55	2168	Naphthalene, 2,3,6-trimethyl-	829-26-5
121	33.100	135, 150, 107	2256	4-Hydroxy-2-methylacetophenone	875-59-2

RT: retention time; RI: retention index.

Table S3. List of VOCs from urine of healthy individuals identified by the optimal non-polar GC-TOF assay.

No.	RT	Top Ions	RI	Compound Name	CAS No./ NIST No.
1	2.111	43, 58, 42	175	Benzaldehyde, 2,4-dinitro-, 2,2-dimethylhydrazone	90535-55-0
2	3.755	43, 72, 57	311	2-Propen-1-ol, 2-methyl-, acetate	820-71-3
3	4.205	69, 74, 131	348	Perfluorooctane	307-34-6
4	4.247	71, 59, 41	351	3-Penten-2-ol	1569-50-2
5	4.596	55, 84, 83	380	2-Ethylacrolein	922-63-4
6	5.956	57, 41, 58	493	Carbonic acid, ethyl 2-propenyl ester	1469-70-1
7	6.117	69, 163, 227	506	Ethyl trifluoromethanesulfonate	425-75-2
8	7.368	96, 67, 95	610	3,4-dimethylfuran	458504
9	7.638	94, 65, 66	632	Carbamic acid, butylmethyl-, phenyl ester	54644-61-0
10	8.082	43, 58, 57	669	Methyl Isobutyl Ketone	108-10-1
11	8.171	84, 55, 39	676	2-Butenal, 2-methyl-, (E)-	497-03-0
12	8.378	57, 43, 100	693	3-Pentanone, 2-methyl-	565-69-5
13	8.434	43, 57, 41	698	2-Pentanone, 3-methyl-	565-61-7
14	9.253	43, 57, 71	766	3-Hexanone	589-38-8
15	9.356	43, 58, 57	774	Pentanal, 2,4-dimethyl-	27944-79-2
16	9.408	81, 110, 32	779	Furan, 2-propyl-	4229-91-8
17	9.470	43, 71, 41	784	3-Pentanone, 2,4-dimethyl-	565-80-0
18	9.634	95, 110, 96	797	2-Furancarboxylic acid, cyclobutyl ester	282759
19	9.664	44, 56, 41	800	Hexanal	66-25-1
20	10.281	81, 53, 112	827	Furan, 2-(methoxymethyl)-	13679-46-4
21	10.369	108, 107, 65	831	Carbamic acid, p-tolyl ester	1850-13-1
22	10.469	57, 85, 41	836	3-Hexanone, 5-methyl-	623-56-3
23	10.542	43, 71, 86	839	2-Pentanone, 3-ethyl-	6137-03-7
24	10.594	57, 41, 114	841	Isobutyl ether	628-55-7
25	10.706	43, 72, 57	846	2-Hexanone, 3-methyl-	2550-21-2
26	10.753	107, 91, 122	848	1,3-Cyclopentadiene, 5,5-dimethyl-1-ethyl-	162257
27	10.840	98, 42, 69	852	2-Furanmethanol	98-00-0
28	10.887	109, 124, 110	854	Ethyl 2-(2-chloroacetamido)-3,3,3-trifluoro-2-(2-fluorobenzylamino)propionate	339352-72-6
29	10.908	41, 55, 69	855	2-Hexenal	505-57-7
30	10.958	58, 43, 82	857	2H-Pyran, 3,4-dihydro-2-methoxy-	4454-05-1
31	11.100	59, 45, 72	864	Ethanedioic acid, dimethyl ester	553-90-2
32	11.302	43, 71, 41	873	4-Heptanone	123-19-3
33	11.592	57, 85, 95	886	3-Heptanone	106-35-4
34	11.639	109, 124, 110	888	1-(3-Methyl-2H-pyrazol-4-yl)ethanone	436162
35	11.669	43, 58, 71	889	2-Heptanone	110-43-0
36	11.782	59, 56, 83	894	N-Chloroacetyl-dl-erythro-O-methylthreonine	214481
37	11.838	104, 103, 78	896	Styrene	100-42-5
38	12.003	109, 124, 110	904	3-Fluoro-o-xylene	443-82-3
39	12.080	79, 94, 48	908	Dimethyl sulfone	67-71-0
40	12.106	111, 112, 97	910	Thiophene, 3,4-dimethyl-	632-15-5

41	12.188	109, 124, 79	914	2-Cyclopenten-1-one, 3,5,5-trimethyl-	24156-95-4
42	12.238	59, 70, 97	916	Butane, 2-methoxy-3-methyl-	62016-49-3
43	12.439	95, 126, 94	927	Methyl 2-furoate	611-13-2
44	12.508	105, 120, 106	930	Benzene, 1-ethyl-4-methyl-	622-96-8
45	12.529	59, 43, 41	931	1,7-Octanediol, 3,7-dimethyl-	107-74-4
46	12.772	43, 72, 57	944	2-Hexanone, 3,4-dimethyl-	19550-10-8
47	13.088	93, 79, 121	960	Bicyclo[2.2.1]heptane, 7,7-dimethyl-2-methylene-	471-84-1
48	13.167	110, 109, 53	964	2-Furancarboxaldehyde, 5-methyl-	620-02-0
49	13.182	91, 92, 119	964	Bicyclo[3.1.0]hex-2-ene, 4-methylene-1-(1-methylethyl)-	36262-09-6
50	13.201	83, 55, 56	965	3-Ethylcyclopentanone	10264-55-8
51	13.435	139, 68, 71	977	2H-Pyran, 2-ethenyltetrahydro-2,6,6-trimethyl-	7392-19-0
52	13.512	119, 59, 91	981	1,3,5-Cycloheptatriene, 3,7,7-trimethyl-	3479-89-8
53	13.560	99, 43, 55	984	3-Pentenoic acid, 4-methyl-	504-85-8
54	13.586	95, 47, 48	985	Cyclohexene, 1-methyl-3-(1-methylethyl)-	13828-31-4
55	13.729	81, 82, 138	992	Furan, 2-pentyl-	3777-69-3
56	13.754	107, 91, 122	994	1-Propanol, 3-(phenylmethoxy)-	4799-68-2
57	13.964	121, 140, 96	1005	Propanedinitrile, [3-(4-methoxyphenyl)-1-methylpropylidene]-	69244-84-4
58	13.980	121, 136, 93	1006	1,3-Cyclopentadiene, 1,2,3,4,5-pentamethyl-	4045-44-7
59	14.037	111, 112, 45	1009	2-Thiophenecarboxaldehyde	98-03-3
60	14.115	91, 119, 134	1013	6,7-Dimethyl-3,5,8,8a-tetrahydro-1H-2-benzopyran	110028-10-9
61	14.149	93, 91, 77	1015	α-Phellandrene	99-83-2
62	14.182	132, 117, 115	1017	Benzene, 1-methyl-2-(2-propenyl)-	1587-04-8
63	14.285	43, 111, 71	1023	7-Oxabicyclo[2.2.1]heptane, 1-methyl-4-(1-methylethyl)-	470-67-7
64	14.337	121, 93, 91	1026	1,3-Cyclohexadiene, 1-methyl-4-(1-methylethyl)-	99-86-5
65	14.354	119, 134, 120	1027	p-Cymene	99-87-6
66	14.469	119, 134, 91	1033	Benzene, 1-methyl-3-(1-methylethyl)-	535-77-3
67	14.493	119, 120, 32	1035	3-Pyridinecarbonitrile, 1,4-dihydro-1-methyl-	19424-15-8
68	14.569	68, 67, 93	1039	Limonene	138-86-3
69	14.637	93, 91, 77	1043	β-Phellandrene	555-10-2
70	14.650	111, 55, 67	1044	4-Methyl-4-vinylbutyrolactone	427992
71	14.664	119, 134, 120	1044	Benzene, 1-ethyl-3,5-dimethyl-	934-74-7
72	15.058	93, 91, 77	1067	γ-Terpinene	99-85-4
73	15.277	59, 43, 94	1079	2-Furanmethanol, 5-ethenyltetrahydro-α,α,5-trimethyl-, cis-	5989-33-3
74	15.420	91, 92, 43	1087	Undecane, 3-phenethyl-1-phenyl-	7225-70-9
75	15.449	132, 117, 115	1089	Benzene, 1-ethenyl-3,5-dimethyl-	5379-20-4
76	15.475	58, 32, 71	1090	Benzo[c]thiophene-1-carboxylic acid, 4,5,6,7-tetrahydro-, (2-dimethylaminoethyl)amide	304411
77	15.481	58, 59, 71	1091	1-(2-Dimethylaminoethyl)-3,3-dimethyldiaziridine	54731-08-7
78	15.551	59, 94, 68	1095	trans-Linalool oxide (furanoid)	34995-77-2
79	15.560	121, 93, 136	1095	Cyclohexene, 1-methyl-4-(1-methylethylidene)-	586-62-9
80	15.624	132, 117, 115	1099	Benzene, 1-methyl-4-(1-methylethenyl)-	1195-32-0
81	15.861	135, 71, 44	1113	3-Adamantan-1-yl-heptan-4-one	296505
82	16.031	119, 134, 91	1124	p-Mentha-1,5,8-triene	21195-59-5
83	16.054	109, 81, 123	1125	1-Allyl-cyclopropanecarboxylic acid, 2,6-di-t-butyl-4-methyl-phenyl ester	108546-69-6

84	16.124	108, 93, 113	1130	1,7,7-Trimethylbicyclo[2.2.1]hept-5-en-2-one	22516-10-5
85	16.207	130, 201, 213	1135	D-Tryptophan	153-94-6
86	16.264	91, 119, 93	1138	cis-Pinen-3-ol	292852
87	16.286	108, 93, 95	1140	α-Campholenal	4501-58-0
88	16.327	95, 138, 123	1142	4-Methoxy-o-phenylenediamine	102-51-2
89	16.459	91, 119, 134	1150	1,3,8-p-Menthatriene	18368-95-1
90	16.782	56, 84, 42	1170	Propane, 1-isocyanato-	110-78-1
91	16.858	59, 339, 94	1175	Phenol, 4,4-butylidenebis[2-(1,1-dimethylethyl)-5-methyl-	85-60-9
92	16.920	108, 150, 149	1179	Benzofuran, 4,5,6,7-tetrahydro-3,6-dimethyl-	494-90-6
93	17.109	95, 81, 71	1190	dl-Menthol	89-78-1
94	17.276	119, 91, 43	1200	Ethanone, 1-(4-methylphenyl)-	122-00-9
95	17.299	137, 69, 107	1202	3,7-Dimethyl-2,3,3a,4,5,6-hexahydro-1-benzofuran #	99606
96	17.395	59, 93, 68	1208	α-Terpineol	98-55-5
97	17.454	73, 154, 99	1212	1,3-Dioxolane, 2-(1,1-dimethylethyl)-	2568-29-8
98	17.492	79, 107, 77	1215	(1R)-(-)-Myrtenal	18486-69-6
99	17.755	159, 174, 160	1232	Naphthalene, 1,2,3,4-tetrahydro-1,1,6-trimethyl-	475-03-6
100	17.799	146, 145, 117	1235	Benzofuran, 4,7-dimethyl-	28715-26-6
101	17.874	73, 122, 32	1240	1,3-Dioxolane, 2-(1-phenylethyl)-	4362-22-5
102	17.931	161, 176, 162	1244	Ethanone, 1-[4-(1,1-dimethylethyl)phenyl]-	943-27-1
103	18.036	137, 69, 109	1251	3,6-Dimethyl-2,3,3a,4,5,7a-hexahydrobenzofuran	70786-44-6
104	18.164	82, 54, 108	1259	(-)-Carvone	6485-40-1
105	18.354	82, 110, 95	1272	2-Cyclohexen-1-one, 3-methyl-6-(1-methylethyl)-	89-81-6
106	18.653	135, 150, 159	1292	Thymol	89-83-8
107	18.667	148, 147, 139	1293	3-Methylbenzothiophene	1455-18-1
108	18.730	161, 176, 162	1297	Benzo[b]thiophene, 7-ethyl-2-methyl-	16587-44-3
109	18.760	109, 81, 41	1299	1-Cyclohexene-1-carboxaldehyde, 4-(1-methylethyl)-	21391-98-0
110	18.837	189, 204, 205	1304	4-tert-Butyl-2,6-dimethylacetophenone	2040-10-0
111	18.909	117, 90, 89	1309	Indole	120-72-9
112	18.968	91, 68, 79	1313	p-Mentha-1,8-dien-7-ol	536-59-4
113	18.988	129, 144, 128	1315	1H-Indene, 4,7-dimethyl-	6974-97-6
114	19.020	121, 93, 152	1317	Benzoic acid, 4-hydroxy-, hydrazide	5351-23-5
115	19.092	150, 135, 71	1322	5-Isopropyl-2-methylphenyl pentanoate	367117
116	19.108	138, 162, 159	1323	3-Methoxy-5-methylphenol	3209-13-0
117	19.121	138, 82, 96	1324	1-Oxaspiro[4.5]dec-6-ene, 2,6,10,10-tetramethyl-	36431-72-8
118	19.183	162, 147, 171	1329	Benzene, 1-methyl-4-[(methylthio)ethynyl]-	17293-64-0
119	19.341	138, 139, 125	1340	(5R,8R)-8-Methyl-5-pentyloctahydroindolizine	117959-79-2
120	19.352	79, 91, 43	1341	1,4-Cyclohexadiene-1-methanol, 4-(1-methylethyl)-	22539-72-6
121	19.485	145, 160, 146	1350	Naphthalene, 1,2,3,4-tetrahydro-1,8-dimethyl-	25419-33-4
122	19.769	173, 85, 188	1370	Benzimidazole, 5-tert-butyl-2-methyl-	5805-62-9
123	19.798	91, 121, 79	1372	Hexane, 1-chloro-5-methyl-	33240-56-1
124	19.893	134, 158, 162	1379	Propionic acid, 3-amino-3-(4-ethylphenyl)-, methyl ester	317077
125	19.902	157, 142, 172	1379	Naphthalene, 1,2-dihydro-1,5,8-trimethyl-	4506-36-9
126	19.925	159, 119, 131	1381	8-Quinolinol, 4-methyl-	3846-73-9

127	20.035	133, 132, 117	1389	4H-1,2,4-Triazol-4-amine, 3-(2,4-dichlorophenyl)-5-(2,4,6-trimethylbenzylthio)-	271709
128	20.120	69, 121, 41	1395	2-Buten-1-one, 1-(2,6,6-trimethyl-1,3-cyclohexadien-1-yl)-, (E)-	23726-93-4
129	20.402	83, 55, 82	1416	Cyclohexane, octyl-	1795-15-9
130	20.408	81, 67, 68	1416	Bicyclo[2.2.2]octane, 2-methyl-	766-53-0
131	20.564	185, 200, 157	1428	Ethanone, 1-(5-methyl-1-phenyl-1H-pyrazol-4-yl)-	6123-63-3
132	20.636	191, 121, 149	1433	Terephthalic acid, 4-isopropylphenyl propyl ester	323632
133	20.900	119, 93, 105	1453	1H-3a,7-Methanoazulene, 2,3,4,7,8,8a-hexahydro-3,6,8,8-tetramethyl-, [3R-(3α,3aβ,7β,8aα)]-	469-61-4
134	20.948	156, 141, 155	1457	Naphthalene, 1,6-dimethyl-	575-43-9
135	21.108	159, 161, 160	1469	2,5-Cyclohexadiene-1,4-dione, 2,6-bis(1,1-dimethylethyl)-	719-22-2
136	21.118	191, 145, 187	1470	2-Pyrrolidinemethanol, 1-(4-nitrophenyl)-	105173-13-5
137	21.130	179, 191, 32	1470	Undecane, 1-(9,10-dihydroanthracen-9-yl)-11-(anthracen-9-yl)-	156039
138	21.320	159, 145, 160	1485	2,5-Dimethoxybenzenesulfonyl chloride	1483-28-9
139	21.510	161, 204, 189	1499	Naphthalene, 1,2,3,5,6,7,8,8a-octahydro-1,8a-dimethyl-7-(1-methylethenyl)-, [1R-(1α,7β,8aα)]-	4630-07-3
140	21.570	94, 204, 161	1504	L-Alanine, N-[N-[N-(3-pyridinylmethylene)-L-valyl]-L-isoleucyl]-, ethyl ester	37580-32-8
141	21.662	191, 57, 192	1511	2,4-Di-tert-butylphenol	96-76-4
142	21.685	189, 204, 161	1513	8-Amino-3-ethyl-7-hydroxy-4-methyl-2H-1-benzopyran-2- one	442128
143	21.707	97, 218, 98	1515	Bicyclo[3.3.1]nonan-1-ol	15158-56-2
144	21.917	183, 198, 149	1531	4-Ethoxycarbonyloxy-3,5-dimethoxybenzoic acid	18780-67-1
145	22.030	161, 134, 204	1540	Naphthalene, 1,2,3,5,6,8a-hexahydro-4,7-dimethyl-1-(1-methylethyl)-, (1S-cis)-	483-76-1
146	22.127	159, 160, 129	1548	Naphthalene, 1,2,3,4-tetrahydro-5,8-dimethyl-1-octyl-	55255-58-8
147	22.140	161, 204, 202	1549	l-Alanine, N-(4-butylbenzoyl)-, tetradecyl ester	314157
148	22.291	220, 205, 221	1561	Propan-2-ol, 1-(4-methoxy-6-methyl-5,6,7,8-tetrahydro-[1,3]dioxolo[4,5-g]isoquinolin-5-yl)-3-(2-methoxyphenyl)-	316543
149	22.391	69, 41, 93	1569	1,6,10-Dodecatrien-3-ol, 3,7,11-trimethyl-, (E)-	40716-66-3
150	22.405	157, 142, 141	1570	4-Isopropyl-6-methyl-1-methylene-1,2,3,4-tetrahydronaphthalene	50277-34-4
151	22.414	93, 81, 59	1571	Cyclohexanemethanol, 4-ethenyl-α,α,4-trimethyl-3-(1-methylethenyl)-, [1R-(1α,3α,4β)]-	639-99-6
152	22.533	220, 205, 137	1580	Benzo[b]dihydropyran, 6-hydroxy-4,4,5,7,8-pentamethyl-	50442-70-1
153	22.548	137, 82, 135	1582	p-Methoxyphenyl-methanediol diacetate	14202-31-4
154	22.901	183, 198, 168	1610	6-Isopropyl-1,4-dimethylnaphthalene	489-77-0
155	22.995	220, 150, 162	1618	Furan, 2,5-diphenyl-	955-83-9
156	23.466	161, 204, 119	1658	Agarospirol	1460-73-7
157	23.501	181, 165, 196	1661	1,1-Biphenyl, 3-(1-methylethyl)-	20282-30-8
158	23.556	189, 204, 133	1665	2-((2S,4aR)-4a,8-Dimethyl-1,2,3,4,4a,5,6,7-octahydronaphthalen-2-yl)propan-2-ol	117066-77-0
159	23.629	159, 220, 160	1671	benzo[b]thiophene-5-carbonitrile	404377
160	23.634	220, 221, 151	1672	1H-Pyrazole, 3,5-diphenyl-	1145-01-3
161	23.641	132, 131, 69	1672	3-(Hydroxyimino)-6-methylindolin-2-one	107976-73-8
162	24.010	183, 198, 168	1704	Naphthalene, 1,6-dimethyl-4-(1-methylethyl)-	483-78-3
163	24.023	93, 119, 69	1705	α-Bisabolol	515-69-5
164	24.113	121, 137, 239	1713	6-Aminonicotinamide	329-89-5

165	24.160	183, 218, 198	1717	Azulene, 1,4-dimethyl-7-(1-methylethyl)-	489-84-9
166	24.279	185, 200, 186	1727	2,4-Imidazolidinedione, 1,3-bis(2-hydroxyethyl)-5,5-dimethyl-	26850-24-8
167	25.492	171, 217, 229	1837	Fenharmane	3851-30-7

RT: retention time; RI: retention index.

Details on data pre-processing for the application in PDAC patients and controls.

In all files, dynamically baseline correction (DBC) was applied in ChromSpace before further analysis. DBC removes contaminant ions (e.g., column bleed or gas ions 28 and 32) and allows better identification when comparing with EI spectra libraries. A pooled QC sample file (.lsc) was deconvolved in ChromSpace and the extracted spectra and retention times (RT) were compared versus NIST 2017 library. Data were filtered by deleting non-linear, irreproducible, or contaminated metabolic features and a list with target mass to charge ratio (m/z) and RTs was compiled and imported to Gavin[1] for peak integration. The number of yielded VOCs after normalization and feature filtering is presented in Table S4.

The extent of the analytical variability in the datasets and its size compared to the biological variability between PDAC patients and controls was assessed by generating principal component analysis (PCA) score plots of the pooled QC and the study samples (Figure 2). Furthermore, the number of linear, reproducible, and non-contaminated VOC features in the pooled QC samples was used as well to assess the quality of the data and the effect of pre-processing steps (Table S4). Several approaches were tested and compared including internal standard (IS) correction[2], total area correction and pooled QC based correction.[3] For internal standard correction, every VOC feature the CV% was normalized with every IS from the same sample and CV% was calculated in each case. The IS giving the lowest CV% was chosen for each feature. For total area correction, every feature of a sample was divided with the sum area of all the features of the same sample. For QC based correction, every feature was normalized with the mean peak area in the first and the last QC of every batch. The rest four QCs of every batch were normalized with the mean peak area in their corresponding batches and were used to calculate the CV% and generate PCA plots to assess the effect of this correction. In the cases that QC based correction was highlighted the optimum pre-processing strategy, the correction was re-calculated utilizing all six pooled QC samples of every batch. While IS and QC correction effect might seem overlapping, since it is impossible to have isotopically labelled analogues of each detected VOC, especially in untargeted studies, QC correction can account for the effects that IS cannot correct due to physicochemical differences between IS and VOCs. IS correction on the other hand, can very effectively correct instrumental drifts, therefore their combination is a valid option and depends on the performance of each dataset. In the polar dataset, IS correction followed by QC based correction yielded the highest number of reliable urinary VOC features (Table S4). In the non-polar dataset, IS correction outperformed all the rest approaches (Table S4). In order to have a consistent strategy in data pre-processing, IS correction followed by QC based correction was adopted for every dataset.

Table S4. Number of reliably measured VOCs after data pre-processing and feature filtering.

Normalization and Filtering	Polar Dataset	Non-polar Dataset
Initial VOC features	261	219
Total area normalization	268	232
QC normalization	294	224
IS correction	264	239
IS correction and QC normalization	294	228

IS: internal standard; QC: quality control.

Table S5. List of urinary VOCs with variable importance projection score (VIP) > 1.5.

No.	Compound Name	CAS No./ NIST No.	VIP	Dataset	Increase/ decrease in PDAC
1	1,1,4,5,6-Pentamethyl-2,3-dihydro-1H-indene	16204-67-4	2.781	Polar	↓
2	3-Pentenoic acid, 4-methyl-	504-85-8	2.517	Polar	↓
3	2-Pentanone	107-87-9	2.397	Polar	↓
4	Silane, diethyldifluoro-	358-06-5	2.393	Polar	↓
5	1,1,1,3,5,5,5-Heptamethyltrisiloxane	1873-88-7	2.340	Polar	↓
6	Benzoyl chloride, 4-pentyl-	49763-65-7	2.334	Polar	↓
7	Hexanal	66-25-1	2.290	Polar	↓
8	2,2,6-trimethyl-cyclohexanone	2408-37-9	2.254	Polar	↑
9	Benzene, (1-methylethyl)-	98-82-8	2.247	Polar	↓
10	Furan-2-carboxamide, N-butyl-N-propyl-	415641	2.238	Polar	↓
11	3-Pentenoic acid, 4-methyl-	504-85-8	2.135	Non-polar	↓
12	Ethyl 2-(2-chloroacetamido)-3,3,3-trifluoro-2-(4-fluorobenzylamino)propionate	339352-65-7	2.098	Non-polar	↓
13	Plastoquinone 3	1168-52-1	2.076	Polar	↓
14	1,3-Benzenediamine, 4-methoxy-	615-05-4	1.998	Polar	↑
15	3-Hexanone	589-38-8	1.996	Polar	↓
16	MethylenedioxyamphetamineN-heptafluorobutyryl deriv.	156572-19-9	1.988	Polar	↓
17	1,3,5,7-Cyclooctatetraene	629-20-9	1.973	Polar	↓
18	p-Cymene	99-87-6	1.966	Polar	↓
19	N-Chloroacetyl-dl-erythro-O-methylthreonine	214481	1.935	Polar	↓
20	Hexanal	66-25-1	1.930	Non-polar	↓
21	Benzene, (1-methylethyl)- (cumene)	98-82-8	1.926	Polar	↓
22	Oxepine, 2,7-dimethyl-	1487-99-6	1.913	Polar	↓
23	Phthalic acid, methyl neopentyl ester	315539	1.904	Polar	↑
24	Levomenol	23089-26-1	1.900	Non-polar	↓
25	o-Isopropenyltoluene	7399-49-7	1.895	Polar	↓
26	3-Isopropylbenzaldehyde	34246-57-6	1.874	Polar	↓
27	4-Isopropyl-6-methyl-1-methylene-1,2,3,4-tetrahydronaphthalene	50277-34-4	1.872	Non-polar	↓
28	2(3H)-Furanone, 5-heptyldihydro-	104-67-6	1.862	Polar	↑
29	Benzene, 1-methyl-4-(1-methylethenyl)-	1195-32-0	1.842	Non-polar	↓
30	2(3H)-Furanone, 5-ethyldihydro-5-methyl-	2865-82-9	1.832	Polar	↓
31	4-Methyl-4-vinylbutyrolactone	427992	1.827	Polar	↓
32	p-Cymene	99-87-6	1.823	Non-polar	↓
33	1-Methoxyadamantane	6221-74-5	1.802	Polar	↓
34	Styrene	100-42-5	1.802	Non-polar	↓
35	5-Hexyn-3-ol	19780-84-8	1.783	Non-polar	↓
36	Naphthalene, 1,2,3,5,6,8a-hexahydro-4,7-dimethyl-1-(1-methylethyl)-, (1S-cis)-	483-76-1	1.761	Non-polar	↑
37	Cyclopentanemethanol, α,α-dimethyl-	1462-06-2	1.755	Non-polar	↓
38	5-Aminoindan	24425-40-9	1.741	Non-polar	↓

39	2-Acetyl-6-methoxynaphthalene	3900-45-6	1.738	Non-polar	↓
40	dl-5-Methyltryptophan	951-55-3	1.732	Non-polar	↑
41	1,2,5-Oxadiazole-3-carboxamide, 4-amino-N-[2-[[(4-fluorophenyl)methyl]amino]ethyl]-	337130	1.718	Polar	↓
42	1-Hexanol	111-27-3	1.717	Polar	↓
43	Pyrrole	109-97-7	1.716	Non-polar	↓
44	Azulene, 1,4-dimethyl-7-(1-methylethyl)-	489-84-9	1.710	Polar	↓
45	(2-Methoxyphenyl)carbamic acid, naphthalen-2-yl ester	305153	1.709	Polar	↑
46	3-Hexanone	589-38-8	1.695	Non-polar	↓
47	Benzene, 1-methoxy-4-(1-methylethyl)-	4132-48-3	1.693	Polar	↑
48	2-Pentanol, 2,3-dimethyl-	4911-70-0	1.682	Non-polar	↓
49	1,3-Dioxolane, 2-methyl-2-pentyl-	4352-95-8	1.672	Polar	↓
50	Ethyl chrysanthemate	97-41-6	1.668	Non-polar	↓
51	Butylphosphonic acid, heptyl neopentyl ester	323126	1.647	Non-polar	↓
52	Acethydrazide, 2-methoxy-2-phenyl-N2-(1-naphthylmethylene)-	351372-17-3	1.640	Non-polar	↓
53	Ethanol, 2-(1-methylethoxy)-	109-59-1	1.634	Polar	↓
54	7-Octen-2-ol, 2,6-dimethyl-	18479-58-8	1.634	Polar	↓
55	m-Cymene, 5-tert-butyl-	29577-19-3	1.627	Non-polar	↓
56	3-Pentenoic acid, 4-methyl-	504-85-8	1.622	Non-polar	↓
57	3,5-Di-tert-butylbenzoic acid	16225-26-6	1.602	Non-polar	↓
58	Sebacic acid, 3,5-dimethylphenyl isobutyl ester	354590	1.593	Polar	↓
59	N-Formyl-d-threo-O-methylthreonine	214695	1.592	Non-polar	↓
60	Tryptamine	61-54-1	1.592	Polar	↓
61	3-Methyl-4-propenyl-oxetan-2-one	194652	1.591	Non-polar	↓
62	Methyl Isobutyl Ketone	108-10-1	1.585	Polar	↓
63	4-Methyl-4-vinylbutyrolactone	427992	1.582	Non-polar	↓
64	Benzene, 1-ethyl-2-methyl-	611-14-3	1.558	Polar	↓
65	Cyclohexene, 1,2-dimethyl-	1674-10-8	1.550	Polar	↓
66	2,3,6-Trifluorobenzyl alcohol, methyl ether	364213	1.543	Non-polar	↑
67	2-Pentanone, 3-ethyl-	6137-03-7	1.530	Non-polar	↓
68	p-Fluoroethylbenzene	459-47-2	1.515	Polar	↓
69	Terbutaline, tetrakis-trifluoroacetyl-	125319-30-4	1.515	Polar	↓
70	Benzene, 1,2,4,5-tetramethyl-	95-93-2	1.510	Polar	↓

References

1. Behrends, V.; Tredwell, G. D.; Bundy, J. G., A software complement to AMDIS for processing GC-MS metabolomic data. *Anal Biochem* **2011,** *415* (2), 206-8.

2. Boysen, A. K.; Heal, K. R.; Carlson, L. T.; Ingalls, A. E., Best-Matched Internal Standard Normalization in Liquid Chromatography–Mass Spectrometry Metabolomics Applied to Environmental Samples. *Anal. Chem.* **2018,** *90* (2), 1363-1369.

3. Alonso, A.; Marsal, S.; Julia, A., Analytical methods in untargeted metabolomics: state of the art in 2015. *Front Bioeng Biotechnol* **2015,** *3*, 23.
